# Back to Roots: Dysbiosis, Obesity, Metabolic Syndrome, Type 2 Diabetes Mellitus, and Obstructive Sleep Apnea—Is There an Objective Connection? A Narrative Review

**DOI:** 10.3390/nu16234057

**Published:** 2024-11-26

**Authors:** Diana Cristina Protasiewicz-Timofticiuc, Diana Bădescu, Maria Moța, Adela Gabriela Ștefan, Adina Mitrea, Diana Clenciu, Ion Cristian Efrem, Maria Magdalena Roșu, Beatrice Elena Vladu, Theodora Claudia Gheonea, Eugen Moța, Ionela Mihaela Vladu

**Affiliations:** 1 Department of Diabetes, Nutrition and Metabolic Diseases, County Clinical Emergency Hospital of Craiova, 200642 Craiova, Romania; diana_protasiewicz@yahoo.com (D.C.P.-T.); drdianabadescu@gmail.com (D.B.); 2 Doctoral School, University of Medicine and Pharmacy of Craiova, 200349 Craiova, Romania; mmota53@yahoo.com (M.M.); eugenmota@yahoo.com (E.M.); 3 Calafat Municipal Hospital, 205200 Calafat, Romania; adela.firanescu@yahoo.com; 4 Department of Diabetes, Nutrition and Metabolic Diseases, Faculty of Medicine, University of Medicine and Pharmacy of Craiova, 200349 Craiova, Romania; theodora.gheonea@umfcv.ro (T.C.G.); ionela.vladu@umfcv.ro (I.M.V.); 5 Department of Medical Semiology, Faculty of Dentistry, University of Medicine and Pharmacy of Craiova, 200349 Craiova, Romania; cristian.efrem@umfcv.ro; 6 Department of Diabetes, Nutrition and Metabolic Diseases, Faculty of Midwives and Nursing, University of Medicine and Pharmacy of Craiova, 200349 Craiova, Romania; maria.rosu@umfcv.ro; 7 Faculty of Medicine, University of Medicine and Pharmacy of Craiova, 200349 Craiova, Romania; beatricevladu75@gmail.com

**Keywords:** gut microbiome, dysbiosis, metabolic syndrome, obesity, cardiovascular disease, obstructive sleep apnea

## Abstract

In recent decades, it has become clear that the gut is more than just a digestive organ; it also functions as an immune organ with regulatory capabilities and acts as a “second brain” that influences brain function due to the presence and regulatory roles of the gut microbiota (GM). The GM is a crucial component of its host and significantly impacts human health. Dysbiosis, or microbial imbalance, has been closely linked to various diseases, including gastrointestinal, neurological, psychiatric, and metabolic disorders. The aim of this narrative review is to highlight the roles of the GM in maintaining metabolic health. Sleep is a vital biological necessity, with living organisms having evolved an internal sleep–wake rhythm that aligns with a roughly 24 h light/dark cycle, and this is known as the circadian rhythm. This cycle is essential for tissue repair, restoration, and overall optimal body functioning. Sleep irregularities have become more prevalent in modern society, with fast-paced lifestyles often disrupting normal sleep patterns. Urban living factors, such as fast food consumption, shift work, exposure to artificial light and nighttime noise, medications, and social activities, can adversely affect circadian rhythms, with dysbiosis being one of the many factors incriminated in the etiology of sleep disorders.

## 1. Introduction

In recent decades, it has become clear that the gut serves not only as a digestive organ but also plays critical roles as an immune organ involved in immune regulation and as a “second brain” influencing brain function, thanks to the presence and regulatory roles of the gut microbiota (GM).

In the human microbiota community, the GM is the predominant component, comprising approximately 10^13^–10^14^ microorganisms, including bacteria, archaea, viruses, and protozoa, and collectively possessing 50 to 100 times more genes than the human genome [1,2].

The gut microbiome interacts with its host, leading to a symbiosis that is highly sensitive to shifts in both the internal and external environment and factors such as infections, medications, lifestyle changes (diet, exercise, smoking, etc.), and the host’s immune response, with these being potential triggers for various responses. It plays a crucial role in sustaining normal gut physiology by promoting food digestion, supporting the maturation of its host’s immune system, and maintaining the integrity of the gut epithelial barrier [3,4].

Taxonomically, the bacteria that colonize the gut are classified into phyla, classes, orders, families, genera, and species, with the Bacteroidetes and Firmicutes phyla comprising 90% of the bacterial populations in the human gut [5], followed by *Actinobacteria*, *Clostridium*, *Proteobacteria*, and *Verrucomicrobia*. Beneficial bacterial families such as *Lactobacillaceae*, *Bifidobacteriaceae*, *Ruminococcaceae*, and *Clostridiales*, part of the Bacteroidetes phylum, serve as primary producers of short-chain fatty acids (SCFAs), molecules which are crucial for maintaining gut homeostasis. Furthermore, SCFAs play a vital role in host–microbe interactions, acting as mediators linking the gut microbiota with brain function [6].

Analyzing the last history dates published in literature, we found out that the GM is an ancient system, going a long way back before humans and making it a key player in the wellbeing of the gastrointestinal tract.

Even more interesting is the fact that the has brain established a unique connection with the GM via a sophisticated and bidirectional communication network that is also known as the gut–brain axis (BGMA) [6,7,8,9,10,11,12].

This amazing discovery was presented as a novel theory by advanced modern medicine studies, which enlightened that many gastrointestinal peptides are also present in the brain. Conversely, certain neurotransmitters are found in the gut [9,13].

So, the BGMA has thus emerged as a demanding pathway connecting the brain and gut, with its balance being essential to the symbiotic relationship between the GM and its host. Generally, the BGMA operates primarily through the following three pathways: the immune system, neuroendocrine system, and vagus nerve [12,13,14,15].

Another interesting fact is that the latest medical studies have shown a new possible axis, the gut–cardiovascular axis, emphasizing the crucial role of the gut microbiota in regulation [16].

## 2. Gut Dysbiosis: An Important Player in Metabolic Health

Lifestyle and dietary habits play crucial roles in maintaining “the internal biological clock”, which is closely linked to “the regulation of metabolic functions in the human body”. Numerous biological processes, including gene activation and deactivation, as well as the initiation of complex metabolic systems that release hormones, digestive enzymes, and metabolites, are regulated by the internal clock in a highly synchronized manner.

**Figure 1:**
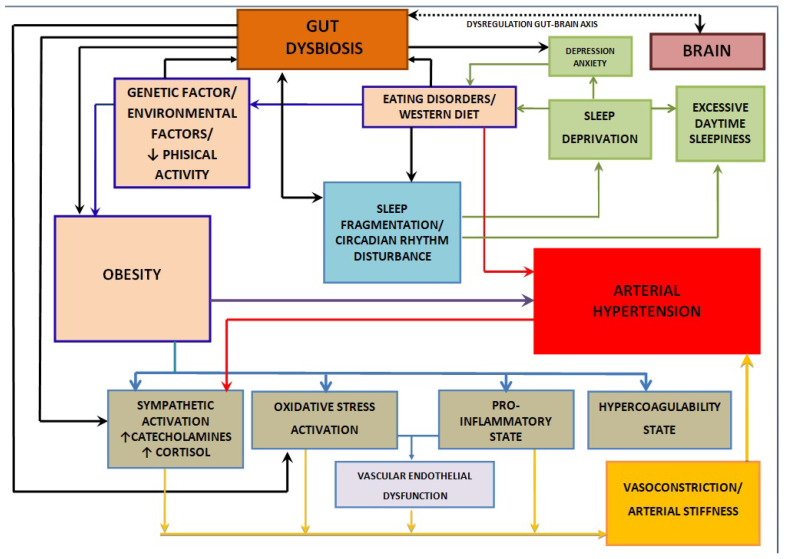
Mechanisms associated with gut dysbiosis, obesity, and arterial hypertension. ↑ Increased level; ↓ Decreased level.

**Figure 2:**
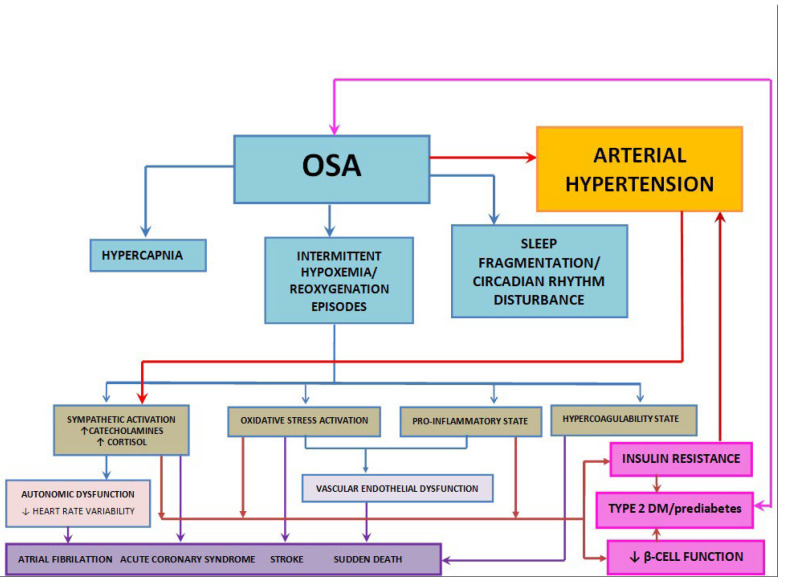
Mechanisms associated with OSA, insulin resistance, and cardio-cerebrovascular events. ↑ Increased level; ↓ Decreased level.

**Figure 3:**
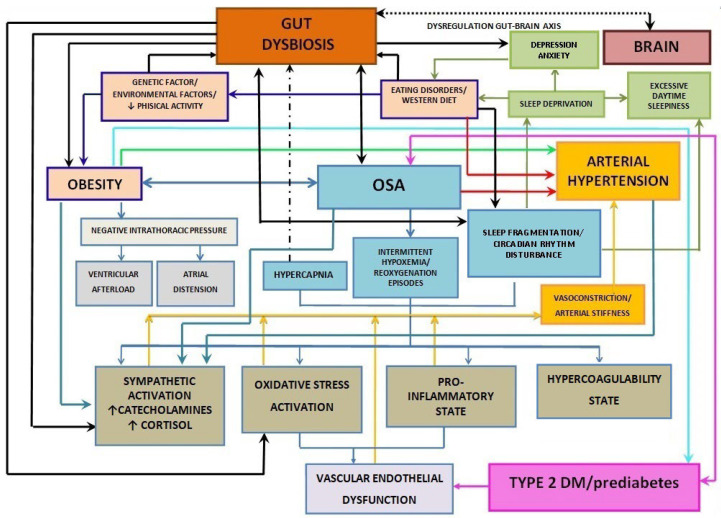
Mechanisms associated with gut dysbiosis, obesity, T2DM/prediabetes, OSA, and arterial hypertension. ↑ Increased level; ↓ Decreased level.

Growing evidence has indicated that the GM is linked to the onset and progression of various diseases, including metabolic disorders, autoimmune conditions, and tumors [17,18,19,20]. Gut dysbiosis, which is defined as a shift in the gut microbiome’s composition, is often marked by reduced microbial diversity, with an increase in the Firmicutes to Bacteroidetes ratio (F:B), as well as a decline in *Bifidobacterium* levels. This imbalance has been linked to various disorders, including obesity, insulin resistance (IR), cardiovascular diseases (CVDs), autoimmune conditions, neurodegeneration, and obstructive sleep apnea (OSA) [21,22,23,24,25,26]. These associations are summarized in Figure 1, Figure 2 and Figure 3.

## 3. The Role of the Gut Microbiota in Sleep Disorders

Nowadays, developments in the latest human technological innovations have led to significant changes in environmental conditions (overeating, unhealthy dietary choices, a lack of or decreased physical activity, and an increased sensitivity response to high psychological stress). These changes seem to have greater effects on wellbeing, inducing poor quality of life and a long-term negative impact on life expectancy [27,28,29,30,31,32,33,34,35,36].

In recent decades, dietary patterns in Westernized countries have shifted dramatically, with a rise in the consumption of calorie-dense, ultra-processed foods that are low in fiber but high in saturated fats, salt, and refined carbohydrates. This subtle but sustained shift has contributed to various negative health outcomes, such as obesity, metabolic syndrome (MetS), type 2 diabetes (T2 D), gestational diabetes mellitus, CVDs, and sleep apnea [37,38,39]. In addition to these factors, chronic sleep deprivation promoted by the modern Western lifestyle is also involved in a range of extremely serious physical and mental health issues [40].

It is obvious that first-line management involves straightforward lifestyle advice. While physical exercise, diet recommendations, and stress release techniques are specifically addressed, sleep is notably absent, although sleep is a fundamental pillar of lifestyle medicine and a fundamental physiological need [40,41].

Normal sleep is characterized by physiological parameters identified through sleep studies, with sleep efficiency being the most crucial. Sleep efficiency, defined as the ratio of total sleep time to time spent in bed and expressed as a percentage, is considered normal when it falls between 85% and 90% [42]. Sleep is an essential biological necessity, and all living organisms have developed an internal sleep–wake cycle that aligns with a roughly 24 h light/dark cycle, which is known as the circadian rhythm or circa diem in Latin, which means approximately one day [41,43]. This rhythm is vital for tissue repair, restoration, and the overall proper functioning of the body [6,44].

Technology has enabled humans to alter their light/dark cycles, as seen with overnight shift work and international travel. However, the human body, along with its “central and peripheral circadian clocks”, has evolved to function according to the natural light/dark cycle that aligns with the earth’s 24 h rotation [45].

The American Academy of Sleep Medicine (AASM) and the Sleep Research Society (SRS) have recommended that adults between 18 and 60 years of age get at least seven hours of sleep each night, while the National Sleep Foundation (NSF) suggests for adults aged 18–64 a period of seven to nine hours of sleep, and in the case of those aged 65 and older, a slightly shorter period of sleep of seven to eight hours per night is suggested [46,47].

The period of sleep has the following two major phases: non-rapid eye movement (NREM) sleep and rapid eye movement (REM) sleep, each serving distinct roles, but of real interest is NREM sleep because it supports tissue repair and immune function [6].

Due to a misalignment between a person’s biological sleep schedule and their 24 h physical, social, and environmental cycles, circadian rhythm sleep disorders can occur. A variety of factors such as prolonged exposure to artificial light, increased nighttime noise, shift work, fast food preferences, the use of certain medications, and other social activities in contemporary modern urban life can disrupt an individual’s circadian rhythm [48,49].

The central circadian clock, located in the suprachiasmatic nucleus (SCN) of the anterior hypothalamus, regulates the sleep–wake cycle by responding to light-dark cues from the environment. Light detected by the retina affects the pineal gland’s melatonin production, which helps initiate and maintain sleep [50]. Circadian rhythms influence nearly all bodily processes, with peripheral clocks found throughout the body [50]. It is surprising and very interesting that the gastrointestinal tract is the largest source of melatonin because melatonin is generated by enterocromaffin cells at levels 400 times higher than the amount produced by the pineal gland [51]. The latest medical studies have suggested the key role played by this hormone in modulating the gut microbiota [52].

As understanding of the brain–gut axis—a two-way communication pathway between the brain and gut—expands, the roles of the GM in sleep have gained significant attention. Evidence has indicated that the GM is vital for maintaining a typical normal sleep physiology. Conversely, abnormal sleep patterns and durations have been shown to impact the diversity, composition, and function of the GM via the brain–gut microbiota axis (BGMA) [6]. What is extremely interesting is the fact that certain substances within the BGMA have a dual role, on one hand as neurotransmitters involved in the process of the brain’s regulation of sleep and on the other hand as endocrine hormones that affect the intestines [9].

Sleep is a reversible physiological process that takes place in the central nervous system (CNS) and is influenced by compelling shifts in brain neuropeptides. Research has shown that several neurotransmitters, including dopamine, glutamate, norepinephrine, serotonin (5-hydroxytryptamine, 5-HT), and γ-aminobutyric acid (GABA), play roles in promoting wakefulness [53]. Additionally, factors such as inflammatory cytokines and the hypothalamic–pituitary–adrenal (HPA) axis have been linked to sleep physiology [54,55].

Revolutionary studies have revealed that the amazing connection between the gut microbiome and the brain’s sleep regulation mechanisms is a complex network of neuroendocrine, immune, and metabolic pathways, and it has a crucial role in enabling an understanding of the relationship between sleep disorders and MetS.

## 4. Gut Dysbiosis—The Physiopathological Pathways Linked to Metabolic Syndrome

Over recent decades, due to human technological innovations, environmental conditions have changed dramatically. Diets high in processed foods and refined carbohydrates, a sedentary lifestyle, and poor sleep appear to highly impact human–microbe relationships. These changes have been linked to a decreased microbiome composition and diversity, elevating inflammation markers and metabolic disease risk, and they are likely to promote the development of MetS.

The global prevalence of MetS, which is a cluster of harmful conditions (e.g., IR, abdominal obesity, and hypertension [56]), is rising swiftly, with estimates indicating that over one billion people worldwide are at increased risk of higher mortality and disease rates due to this syndrome [57].

Gut dysbiosis in the gut microbiota reduces microbial richness and diversity, allowing commensal and opportunistic pathogens to dominate over beneficial bacteria. Alterations in the *Firmicutes*/*Bacteroidetes* and *Prevotella*/*Bacteroides* ratios are commonly observed in obesity, indicating that the microbiota could potentially predict increased body mass index (BMI) and fat mass.

Over recent decades, obesity has become increasingly widespread, and recent global estimations have indicated that nearly 3.3 billion adults could be affected by high BMIs by 2035 [58]. This condition is driven in part by a combination of genetic and behavioral factors, including higher consumption of calorie-dense and processed foods and more sedentary lifestyles [59].

Sleep deprivation results in suppressed leptin levels and elevated ghrelin levels compared to adequate sleep. These hormonal changes induced by sleep deprivation would be expected to stimulate appetite [59].

Many defining characteristics of metabolic diseases such as obesity, T2 D, CVD, and liver steatosis [60,61] suggest a causative role of gut microbial metabolism [62].

The key role of the GM’s involvement in the human physiology of nutrition, metabolism, and immune function is well established, making it a vital endocrine–metabolic organ [63].

To be able to more clearly understand the key role of dysbiosis in the development of metabolic disorders, the latest studies have proposed several physiopathological mechanisms that could be involved. The first mechanism involves the innate immune system’s contact-mediated response to specific microbial molecular patterns, such as toll-like receptors (TLRs) and NOD-like receptors (NLRs), leading to local low-grade inflammation. The second mechanism is an indirect effect of microbial metabolism that impacts intestinal epithelial integrity by disrupting the tight junctions between epithelial cells. Increased gut permeability, also known as “leaky gut”, along with microbial dysbiosis, and especially gut dysbiosis, are found to be involved in metabolic diseases like obesity and diabetes [64].

Disruptions in intestinal homeostasis and increased gut permeability are believed to allow microbial-derived compounds such as lipopolysaccharides (LPS), endotoxins, and flagellins to enter the bloodstream, contributing to systemic inflammation. The reduction in microbial diversity and richness and the high prevalence of pathogenic bacteria, often seen in metabolic disorders, are thought to play a role in low-grade metabolic inflammation, fueling pathological processes like IR and vascular dysfunction. The hallmark of metabolic disorders is the presence of low-grade systemic inflammation, characterized by elevated circulating levels of inflammatory cytokines (such as TNF-α, IL-1β, IL-6, and IL-17) and an increased immune cell presence in insulin-dependent tissues, with all of these processes occurring without any tissue damage [65].

Le Chatelier et al. were the first to show that individuals with low microbial gene richness were marked by adiposity, IR, and dyslipidemia, with gene counts correlating with metabolic factors such as insulin levels and IR [66]. Further evidence came from a recent study by Asnicar et al. on the PREDICT1 cohort, which confirmed that specific gut microbiome compositions were correlated with a broad range of cardiometabolic blood markers (e.g., fasting, postprandial glycemia, and lipemia), and pro-inflammatory markers [67].

## 5. The Relationship Between Gut Dysbiosis and OSA—A Central Link in the Continuum of Sleep Apnea, Insulin Resistance, and Cardiovascular Disease

OSA is an escalating global health concern associated with significant healthcare costs, particularly as studies have shown it to be a risk factor for several non-communicable diseases [68].

OSA is a health condition characterized by the repeated partial or complete blockage of the upper airway during sleep. This leads to fluctuations in intrathoracic pressure, intermittent drops in blood oxygen levels, and disrupted sleep, all of which diminish quality of life and significantly affect both physical and mental health. Additionally, OSA is linked to both functional and structural metabolic disorders, making it a significant worldwide and real public health concern [69,70,71,72,73,74].

The prevalence of OSA is on the rise, partly due to the global increase in obesity, which is a well-known cause of OSA [75]. Approximately 35% of patients with primary hypertension have OSA, with the prevalence rising to approximately 80% in those with drug-resistant hypertension [76].

Nowadays, OSA is an underdiagnosed disorder that causes sleep disruption, sleep fragmentation, intermittent hypoxia (IH), sympathetic hyperactivity, and very important disturbances in physiological homeostasis. OSA patients typically suffer from poor and disrupted sleep, which can lead to fatigue, excessive daytime sleepiness, and difficulty concentrating. OSA significantly exacerbates the risk of metabolic dysfunction and AVD. The latest studies have revealed the fact that gut dysbiosis is involved in OSA pathogenesis [77].

The apnea–hypopnea index (AHI), which measures the number of apneas and hypopneas during sleep, is the most-used clinical parameter to assess the severity of OSA [78]. Numerous studies have explored how the gut microbiome is affected by different stages of OSA severity. OSA severity is linked to lower overall microbial diversity and more homogeneous compositions of microbial communities [79]. Thus, more severe cases of OSA may be associated with a higher abundance of *Fusobacterium* in the gut. This bacterial species is also found in greater numbers in patients with generalized anxiety disorder. A meta-analysis has indicated that anxiety impacts approximately one-third of individuals with OSA, suggesting a potential link between *Fusobacterium* and this association [80,81,82], an aspect of great importance to be considered in the management of an OSA patient.

The interrelation between gut dysbiosis and OSA may be largely explained by the following intricate mechanisms: (1) a disrupted gut barrier, which leads to a proinflammatory state; (2) an alteration to the immune and neuroendocrine systems (neuroinflammation); (3) SCFAs; and (4) the vagus nerve pathway.

### 5.1. A Disrupted Gut Barrier, a Proinflamatory State, and Neuroinflammation

Recent evidence has suggested that inflammation caused by OSA plays a significant role in many of its associated cardiovascular effects [83,84,85]. Also, the latest studies have demonstrated that the gut microbiota significantly influence host immune function [86]. Microbes and their products affect the host’s immune response, with some signals promoting inflammation and others reducing it [87]. Moreover, the impact of microbes on the host’s immune response extends beyond the gut wall. Microbial signals that enter the bloodstream can influence immune cells in distant parts of the body [88,89]. It seems like gut dysbiosis can trigger inflammation in the gut, and the immune cells originating there can migrate to distant tissues, including the brain [90]. So, gut dysbiosis has been shown to influence brain homeostasis and neuroinflammation [91].

Considering the link between gut dysbiosis and host inflammation, along with the role of neuroinflammation in the development of OSA-induced hypertension, OSA triggers gut inflammation, which, in turn, contributes to neuroinflammation and hypertension.

Cell tracking studies have revealed that OSA increases the movement of TH1, TH2, and TH17 cells from Peyer’s patches in the small intestine to the mesenteric lymph nodes (MLNs), spleen, and brain. These findings have collectively shown that OSA triggers a proinflammatory response in both the gut and brain, contributing to the development of OSA-induced hypertension.

OSA is linked to the disruption of the gut barrier [92]. After a disruption to the gut barrier, bacteria and bacterial antigens can cross the epithelium, leading to the activation and migration of antigen-presenting cells to the mesenteric lymph nodes (MLNs), T-cell activation, and peripheral inflammation. Consequently, we have observed significant increases in TH1 cells, macrophages, and TNF-α+ cells in the spleen, as well as increased TH1 and TNF-α+ cells in the aorta. OSA causes an efflux of T lymphocytes from the Peyer’s patches in the small intestine, which then migrate to the MLNs, spleen, and brain. Thus, in the pathogenesis of hypertension in patients, neuroinflammation plays a key role [93,94,95,96]. Thus, neuroinflammation is a hallmark of OSA-induced hypertension [97].

Recent medical studies investigating changes in the gut microbiome’s diversity in humans with arterial hypertension using ultra-modern genetic methods have shown reductions in *Roseburia* spp., *Faecalibacterium prausnitzii*, and the butyrate-producing bacteria *Odoribacter* and increases in *Prevotella*, *Klebsiella* spp., *Streptococcus* spp., and *Parabacteroides merdae* [98,99,100,101].

### 5.2. Gut Dysbiosis Leads to Disrupted SCFAs Metabolism—The Impact Upon OSA

SCFAs are the metabolic end products of the microbial fermentation of dietary fibers. The human gut lacks the enzymes needed to break down certain foods, specifically, complex carbohydrates in dietary fiber. However, specific anaerobic bacteria in the cecum and large intestine can ferment these fibers into various by-products, with SCFAs being the most extensively studied. The three predominant SCFAs in the human colon are acetate, propionate, and butyrate, which are typically present in an approximate molar ratio of 60:20:20, respectively. Two main pathways (G-protein-coupled receptor activation and/or histone deacetylaze inhibition) are used by these three SCFAs to stabilize the gut epithelial barrier, increase the protective mucus layer, balance cytokine secretion, adjust antibody secretion, and alter T-lymphocyte populations, thus having a local effect on the gut and one at a distance upon other tissues and organs throughout the circulatory system [92].

Lower levels of acetate and butyrate have been observed in individuals with OSA-associated hypertension, with these disruptions in SCFA metabolism thought to play a central role in OSA pathogenesis. Hypoxia-induced oxidative stress may drive metabolic alterations in OSA patients. A recent study in human subjects found high levels of branched-chain amino acids (BCAAs) such as valine, leucine, and isoleucine [102]. SCFAs, such as butyrate, have an impact on the genes involved in regulating circadian rhythms and sleep [103,104,105]. Recent studies have linked OSA to a reduction in butyrate-producing bacteria, such as *F. prausnitzii* [106,107].

### 5.3. Gut Dysbiosis and Central Nervous System Activity—The Vagus Nerve Pathway

Additionally, the gut microbiota may affect sleep through the vagus nerve, which contains both efferent and afferent neurons [108]. Large-scale medical studies on animals have demonstrated that the gut microbiome can influence central nervous system activity by stimulating vagal afferents throughout various mechanisms. The most important ones include the release of butyrate and neurotransmitters (e.g., γ-aminobutyric acid and serotonin) [109].

Research on epileptic patients has indicated that vagal stimulation can influence both breathing patterns during sleep and overall sleep quality [110,111,112]. Sleep deprivation is extremely frequent in both epilepsy and OSA, and it is associated with behavioral abnormalities and important metabolic changes that can have a greatly negative impact upon the gut microbiota. For instance, the SCFAs that are microbial metabolites play a role in sleep regulation and maintaining circadian rhythms. Gut dysbiosis, along with the decreased production of SCFAs, may cause sleep disturbances [113].

Inadequate sleep, often linked to OSA, has been shown to induce behavioral and metabolic changes that adversely affect the diversity and composition of the gut microbiome [14,114,115,116,117,118], and correspondingly, gut dysbiosis may contribute to sleep disturbances related to OSA. For example, gut dysbiosis can result in digestive problems like constipation and bloating, followed by abdominal pain and changes in bowel movements, which often can disrupt sleep, especially if they occur at night [119].

Sleep disruption, particularly due to OSA, can have significant systemic effects. OSA is characterized by intermittent hypoxia and hypercapnia. The intermittent hypoxia associated with OSA can cause changes in the gut microbiota, resulting in the dysregulation of the gut–brain axis [120].

## 6. Gut Dysbiosis as an Atherosclerotic Promoter in OSA

OSA is independently associated with an elevated risk of cardiovascular events like myocardial infarction, stroke, and cardiovascular mortality, mainly through the promotion of severe atherosclerosis (ATS).

The gut microbiota likely influence ATS through the following three mechanisms: (i) bacterial infections that can activate the immune system, leading to a detrimental inflammatory response that exacerbates plaque progression followed by rupture; (ii) changes in cholesterol and lipid metabolism driven by the gut microbiota that impact the development of ATS; and (iii) microbial metabolites that can have either positive or negative effects on ATS [121]. Recent studies have suggested that these three mechanisms are likely to play a significant role in ATS induced by OSA [122,123].

Intermittent hypoxia, a hallmark of OSA, alters the gut microbiota and metabolite profiles. These changes in the gut environment likely contribute to ATS development by affecting gut permeability, inflammatory responses, microbial metabolites like trimethylamine (TMA) and bile acid, and lipid metabolism [124].

In recent years, numerous medical studies have been conducted all over the world to discover how changes in the microbiome’s diversity lead to various cardiovascular diseases. Thus, using modern genetic methods, like metagenomics sequencing, terminal restriction fragment length polymorphism, and 16S sequencing in humans with ATS and coronary artery disease, researchers have identified increases in *Streptococcus*, *Escherichia*, Lactobacillales, *Collinsella*, *Curvibacter*, Burkholderiales, *Propionibacterium*, and *Ralstonia* and decreases in *Bacteroides*, *Prevotella*, *Roseburia*, *Eubacterium*, *Clostridium*, *Faecalibacterium*, *Burkholderia*, *Corynebacterium* and *Sediminibacterium*, *Comamonadaceae*, *Oxalobacteraceae*, *Rhodospirillaceae*, *Bradyrhizobiaceae*, and *Burkholderiaceae* [125,126,127,128,129,130,131]. 

## 7. Discussion and Conclusions

Links and active interactions between gut dysbiosis induced by the modern Western diet, obesity, MetS, T2 D, OSA, CVD, and tumors are made more and more often in the current medical literature. Dietary choices influence microbial composition and metabolite production, leading to both improvements and deteriorations in MetS and sleep homeostasis.

The emergence and progression of CVDs lies under the umbrella of various risk factors and pathological mechanisms like low-grade inflammation, dyslipidemia, insulin resistance, and diabetes mellitus [132,133].

Knowing that the GM acts as a virtual endocrine–metabolic organ that is able to produce metabolites that are bioactive (TMA/TMAO, SCFA, and bile acids), with great impact on its host’s wellness or disease, we are looking into the future with great expectations as the GM and its’ complex metabolic pathways become more and more attractive as potential targets for CVD interventions [132,134].

A new and promising therapeutic approach is modulating the GM through diet and certain lifestyle changes, which might be used as a tool for lowering the risk of some CVDs [135].

Being responsible for more than 50% of all cardiovascular deaths, atherosclerosis is the leading cause of chronic heart disease and stroke. The “newest rising star” to be considered in the pathogenesis of CVDs is gut dysbiosis [136]. Although current studies have their own limitations, understanding the mechanisms involved in the gut–heart axis may, in the near future, offer personalized intervention therapies for cardiovascular health despite the objective concerns about the long-term consequences and safety of microbiota modulation therapy [137].

It is extremely crucial to keep the right balance between the nonpathogenic and pathogenic microorganisms residing in the human gut. Numerous studies have tried to analyze different types of diets and their impacts upon the microbiota composition in the prevention of CVDs, the most efficient ones being the Mediterranean diet (the ingestion of plant foods and the moderate consumption of fish, seafood, dairy products, red wine, and olive oil) and plant-based diets (seeds, cereals, fruits, berries, nuts, and vegetables), emphasizing the vital role of consuming fiber and bioactive compounds [136]. Besides sleep and diet, another key player in healthy lifestyle choices is represented by regular moderate physical activity. Nowadays, from a more artistic point of view, these are the three main known actors on the everchanging microbiota stage, although the use of probiotics and fecal microbiota transplant might gain their places in the near future.

A limitation of this paper is the fact that it is a narrative review, and therefore, it cannot provide statistical analyses like a systematic review or a meta-analysis, with the connections drawn between gut dysbiosis and health outcomes being largely correlational.

All the evidence presented emphasizes the importance of adopting healthy dietary patterns rich in dietary fiber to enhance the abundance of key beneficial bacteria in the gut microbiota of individuals with MetS that are also associated (or not) with sleep disorders, as this suggests a potential link between the gut microbiota and the metabolic alterations commonly observed in MetS and sleep disorders. In this regard, nonetheless, further extensive observational and more mechanistic studies are needed not only to confirm but also validate these findings.
